# Effectiveness and feasibility of early physical rehabilitation programs for geriatric hospitalized patients: a systematic review

**DOI:** 10.1186/1471-2318-13-107

**Published:** 2013-10-10

**Authors:** Nienke M Kosse, Alisa L Dutmer, Lena Dasenbrock, Jürgen M Bauer, Claudine JC Lamoth

**Affiliations:** 1 University of Groningen, University Medical Center Groningen, Center for Human Movement Sciences, Groningen, The Netherlands; 2 Geriatrics Center Oldenburg, Carl von Ossietzky Universität Oldenburg, Oldenburg, Germany

**Keywords:** Acute care, Rehabilitation, Hospital, Aged, Functional outcomes, Feasibility, ADL, Physical performance

## Abstract

**Background:**

Old adults admitted to the hospital are at severe risk of functional loss during hospitalization. Early in-hospital physical rehabilitation programs appear to prevent functional loss in geriatric patients. The first aim of this review was to investigate the effect of early physical rehabilitation programs on physical functioning among geriatric patients acutely admitted to the hospital. The second aim was to evaluate the feasibility of early physical rehabilitation programs.

**Methods:**

Two searches, one for physical functioning and one for feasibility, were conducted in PubMed, CINAHL, and EMBASE. Additional studies were identified through reference and citation tracking. To be included articles had to report on in-hospital early physical rehabilitation of patients aged 65 years and older with an outcome measure of physical functioning. Studies were excluded when the treatment was performed on specialized units other than geriatric units. Randomized controlled trials were included to examine the effect of early physical rehabilitation on physical functioning, length of stay and discharge destination. To investigate feasibility also non randomized controlled trials were added.

**Results:**

Fifteen articles, reporting on 13 studies, described the effect on physical functioning. The early physical rehabilitation programs were classified in multidisciplinary programs with an exercise component and usual care with an exercise component. Multidisciplinary programs focussed more on facilitating discharge home and independent ADL, whereas exercise programs aimed at improving functional outcomes. At time of discharge patients who had participated in a multidisciplinary program or exercise program improved more on physical functional tests and were less likely to be discharged to a nursing home compared to patients receiving only usual care. In addition, multidisciplinary programs reduced the length of hospital stay significantly. Follow-up interventions improved physical functioning after discharge. The feasibility search yielded four articles. The feasibility results showed that early physical rehabilitation for acutely hospitalized old adults was safe. Adherence rates differed between studies and the recruitment of patients was sometimes challenging.

**Conclusions:**

Early physical rehabilitation care for acutely hospitalized old adults leads to functional benefits and can be safely executed. Further research is needed to specifically quantify the physical component in early physical rehabilitation programs.

## Background

The rapidly growing population of old adults in Western countries has become a major concern for health care systems. Due to a poorer health status, old adults consume a disproportionate amount of medical care. In some European countries, more than 40% of patients admitted to the hospital for an overnight stay are aged 65 years and older, while their total share of the population is less than 20% [1].

Unfortunately, old adults admitted to the hospital are at severe risk of functional decline, both during hospitalization and after discharge [2,3]. A number of studies found that approximately 33% of the patients have severe functional deterioration at time of discharge compared to their status before hospital admission [3-5]. For patients 90 years or older this number even increases to 63% [4]. Functional decline during and after hospital stay has shown to be an important risk factor for nursing home placement [6,7].

The decline in functional capacity seems to be partly the result of the hospitalization itself, unrelated to diagnostic or therapeutic interventions. Older patients have decreased physiological and functional reserves that make them more vulnerable to the effects of bed rest and decrease in dietary intake, which both are highly prevalent during hospitalization. Due to immobilisation, muscle strength and aerobic capacity tend to decline rapidly. After only ten days of bed rest healthy old adults lose 12-14% of both their VO_2max_ and lower extremity muscle strength [8]. Without any voluntary muscle contractions muscle strength can even decrease by 5% per day [2].

Altogether, functional decline is a common problem that is significantly associated with negative outcomes such as institutionalization, re-hospitalization and subsequent mortality [3]. The primary focus of hospital care is treating acute and chronic illnesses. A physical rehabilitation intervention that may preserve physical function is often not part of the treatment. To preclude a rapid decline in physical function it is important that hospital programs are also directed explicitly towards activating the older patient early after hospital admission. Early physical rehabilitation might help to prevent decline in physical functioning arising from immobility and prolonged bed rest [9].

Over the years, several multidisciplinary and exercise types of early rehabilitation interventions have been studied. Previous studies showed that early rehabilitation programs improved both patient (e.g. physical functioning) and hospital outcomes (e.g. reducing costs) for acute ill geriatric patients [10,11]. However, an important issue not yet addressed in the current literature is the feasibility of in-hospital early exercise programs for acute geriatric patients. To start an early physical rehabilitation program, knowledge of which patient population benefit from the program is required. Furthermore, it is important to know if there will be adverse events during early physical rehabilitation programs in terms of falls or other injuries and what the adherence rate of the patients will be during the treatment sessions. Therefore, the first aim of this review is to evaluate the effects of early physical rehabilitation programs on physical functioning of geriatric patients acutely admitted to a hospital. In this review early physical rehabilitation in acutely ill patients refers to physical therapy, occupational therapy, and physical exercises initiated immediately upon achieving physiologic stability and continued throughout the hospital stay. Such activities start within 1 or 2 days after hospitalization. The second aim of the present review is to evaluate the feasibility of early physical rehabilitation programs in the hospitalized geriatric patients.

## Method

### Search strategy

A systematic literature search was conducted in three electronic databases, PubMed, CINAHL, and EMBASE in August 2013. Keywords used to perform the search were: “aged” or “elderly” or “geriatric”, “hospital” or “hospitalized”, “exercise” or “rehabilitation”, “ADL” or “physical functioning” or “mobility” or “physical performance”, and “acute” or “acutely”. The articles included were randomized controlled trials (RCT), written in English, including participants aged 65 years or older who were acutely admitted to the hospital. The interventions investigated in the articles needed to include a physical exercise part with a physical functioning measure as outcome. Studies were excluded if the included patients required treatment on a specialized unit other than an acute geriatric unit or when the evaluated intervention aimed at a specific disorder or surgical process. Articles about the feasibility of early physical rehabilitation of inpatients were retrieved by adding the keywords “feasibility” or “feasible” or “adherence rate” or “safety” to the keywords mentioned above. The articles about feasibility had the same in and exclusion criteria as the articles addressing physical functioning with the exception that also non randomized controlled trials were included. For the inclusion process title and abstract were examined and when necessary the full article was obtained and read. Additional studies were identified through reference and citation tracking. Two reviewers independently screened title, abstract and full text. Disagreement about inclusion of articles was resolved by discussion and consensus between the two reviewers.

### Data extraction and analysis

Data were extracted against pre-defined categories by two researchers. The data compiled from the studies included information on: study design, characteristics of participants and setting, the intervention and control group treatment, time of assessment, ADL, physical performance, length of stay and discharge destination. Furthermore, the feasibility outcomes were the ability to enrol patients into the rehabilitation program, and the adherence rate and safety of the patients during the therapy sessions. The information extracted from the articles was organized into tables and systematically compared.

### Methodological quality

The methodological quality of the included RCTs was assessed using the Delphi scale [12]. The Delphi scale is a quality assessment tool for RCTs and has shown to be valid and reliable [13]. It consists of 9 different criteria which can be scored positive, negative, or unclear (“yes”, “no”, and “don’t know”). One point was given for each “yes” and zero points for each “no” or “don’t know”, the total quality sore ranged from 0 (low quality) to 9 (high quality).

## Results

### Selected studies

**Figure 1:**
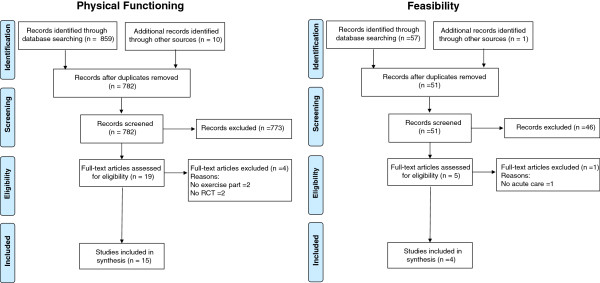
Flowcharts of search results.

The literature search for physical functioning yielded a total of 772 papers (Figure 1). After removing 765 articles based on title and abstract, 9 articles were qualified for full text reading. Four articles were removed after full text reading and ten articles were added after reference checking. The remaining 15 articles, describing 13 studies, were included to this review. The search for feasibility studies yielded 50 papers. After removing 47 articles by title, abstract and full text reading and adding one article after reference checking, four studies were included for the feasibility assessment. The article of Laver et al. [14] was included in both the physical functioning and the feasibility section. PRISMA guidelines were followed in this systematic review [15].

### Methodological quality

In Table 1 the quality scores on the Delphi Scale for the different RCT studies are reported. Total quality scores ranged from 3 to 7 with a median score of 5. The methodological quality was moderate for most studies. Randomization methods and eligibility criteria were clearly defined in all 13 studies. The studies scored particularly low on blinding the assessor, the care provider and the participant. The Delphi scores were good for the concealed treatment allocation, the similarity of the intervention and control groups at baseline and the clarity of the specified eligibility criteria.

**Table 1:** Methodological quality scores on the Delphi scale for each RCT study

**Study**	**Randomized**	**Treatment allocation concealed**	**Groups similar at baseline**	**Eligibility criteria specified**	**Outcome assessor blinded**	**Care provider blinded**	**Patient blinded**	**Variability measures**	**Intention-to-treat analysis**	**Total (max 9)**
Abizanda [16]	1	1	0	1	1	1	1	1	0	7
Asplund [17]	1	1	1	1	0	0	0	0	0	4
Blanc-Bisson [18]	1	0	1	1	0	0	0	1	1	5
Counsell [19]	1	1	1	1	0	0	0	1	1	6
Courtney [20,21]	1	1	1	1	1	0	0	1	1	7
De Morton [22]	1	0	0	1	0	0	1	1	1	5
Jones [23]	1	1	0	1	0	0	0	1	1	5
Landefeld [24]	1	0	1	1	0	0	0	0	0	3
Laver [14]	1	1	1	1	1	0	0	1	1	7
Nikolaus [25]	1	1	1	1	1	0	0	1	0	6
Saltvedt [26,27]	1	1	1	1	0	0	0	1	0	5
Siebens [28]	1	1	1	1	0	0	0	0	1	5
Slaets [29]	1	1	1	1	0	0	0	0	0	4

### Inclusion criteria and patient characteristics

Table 2 summarizes the characteristics of patients, study settings, early physical rehabilitation programs and outcomes of the included RCT studies in this review. The mean age for patients admitted to acute care in the hospital for a general medical condition varied between 78 and 86 years old. The most common reasons for admission were cardiac problems, respiratory problems, gastrointestinal problems, neurological problems, infections and injuries caused by a fall. The living situation of the patients before they were admitted to the hospital varied, patients came from nursing homes and other types of institutionalised care or from the community where they lived alone or with family. However, the studies did not include all patients in the intervention, reasons for exclusion were medical instability [18,22,23], need for specialized care [17,19,22,24], living in nursing homes [19,20,22,23,25,26,28], small survival chance or need for palliative care [18,22,23,25,28], and being diagnosed with an illness causing functional impairment [18,23,28]. Overall, there was a great heterogeneity among the participants between the different studies.

**Table 2:** Setting and study characteristics physical functioning

**Study**	**Population & Setting**	**Intervention**	**Time of assessment**					
				**(I)ADL**	**Physical Performance**	**Mortality (%)**	**LOS (days)**	**Discharge ICF (%)**
**Multidisciplinary care with an exercise component**								
Asplund 2000 [17]	**Intervention** (n=190)	Multidisciplinary team, with physical and occupational therapy. Discharge planning and early rehabilitation.	T0 Admission	BI ≥ 19 points	NA	T1 4.2	5.9^*^	T1 11.6
	Mean age 80.9 years		T1 Discharge	T0 52%		T2 11.1		T2 11.6
	58% female, AGU, UH		T2 3 months post-discharge	T2 44%				
	**Control** (n=223)	General medical unit care		BI ≥ 19 points		T1 2.7	7.3^*^	T1 19.3
	Mean age 81.0 years			T0 44%		T2 7.6		T2 18.4
	63% female, MU, UH			T2 43%				
Counsell 2000 [19]	**Intervention** (n=767) †	Multidisciplinary team, with daily assessment of physical functioning and protocols to improve self-care and mobility. Early discharge planning.	T0 2 weeks pre-admission	ADL decline	PPME score	T1 2.7	6.1	T1 12.9
	Mean age 80 years		T1 Discharge	T1 30%	T1 5.6^*^	T2 9.0		T2 10.3
	60% female, CH		T2 1 months post-discharge	T2 27%		T3 15.9		T3 8.9
			T3 3 months post-discharge	T3 26%		T4 22.6		T4 7.5
			T4 6 months post-discharge	T4 22%		T5 31.4		T5 6.7
			T5 1 year post-discharge	T5 25%				
	**Control** (n=764) †	Usual physician and nursing staff care		ADL decline	PPME score	T1 3.7	6.3	T1 15.6
	Mean age 79 years			T1 34%	T1 5.0^*^	T2 11.3		T2 10.1
	61% female, CH			T2 29%				
				T3 26%		T3 17.4		T3 7.2
				T4 30%				
				T5 30%		T4 22.5		T4 8.0
						T5 29.2		T5 7.3
Landefeld 1995 [24]	**Intervention** (n=327)	Multidisciplinary program, with daily assessment of physical functioning and protocols to improve self-care and mobility	T0 Admission	ADL score:	NA	T1 7.3	7.3	T1 5.8^*^
	Mean age 80.2 years		T1 Discharge	T0 3.0		T2 20.8		T2 13.1^*^
	68% female, MU, CH		T2 3 months post-discharge	T1 3.6^*^				
				T2 4.0				
				IADL score:				
				T0 2.8				
				T1 3.3^*^				
				T2 3.9				
	**Control** (n=324)	Usual care services provided by physicians and nurses		ADL score:		T1 7.4	8.3	T1 11.7^*^
	Mean age 80.1 years			T0 3.0		T2 19.8		T2 18.8^*^
	65% female, MU, CH							
				T2 3.8				
				IADL score:				
				T0 2.8				
				T2 3.8				
Saltvedt 2002 [26,27]	**Intervention** (n=127) †	Interdisciplinary program to prevent complications, with early mobilization, rehabilitation and discharge planning	T0 Admission	ADL dependence	NA	T1 11.8^*^	15^*^	NA
	Mean age 81.8 years		T1 3 months post-discharge	T1 21%		T3 26.8^*^		
	81% female, GU, UH		T2 6 months post-discharge	T2 13%				
			T3 1 year post-discharge	T3 25%				
				IADL dependence				
				T1 46%				
				T2 44%				
				T3 45%				
	**Control** (n=127) †	Usual care		ADL dependence		T1 27.6^*^	7^*^	
	Mean age 82.4 years			T1 12%		T2 33.9^*^		
	84% female, MU, UH			T2 13%				
				T3 23%				
				IADL dependence				
				T1 39%				
				T2 40%				
				T3 44%				
Slaets 1997 [29]	**Intervention** (n=140)	Multidisciplinary program added to the usual care. Geriatrician, physiotherapist and liaison nurse obtained optimal ADL and mobility in 2 hours training a day.	T0 Admission	Improved ADL	Improved mobility	NA	19.7^*^	T2 18^*^
	Mean age 82.5 years		T1 Discharge	T1 61%^*^	T1 48%^*^			
	67% female , MU, CH		T2 1 year post-discharge					
	**Control** (n=97)	Usual care: services provided by physicians and nurses.		Improved ADL	Improved mobility		24.8^*^	T2 27^*^
	Mean age 83.2 years			T1 46%^*^	T1 44%^*^			
	75% female, MU, CH							
**Usual care programs with an exercise intervention**								
Abinzanda 2011 [16]	**Intervention** (n=198)	Conventional treatment plus occupational therapy: 5 days per week, 30 - 45 min a day	T0 Admission	55.6% improved ≥ 10 BI points	NA	T1 7.6	9.1	NA
	Mean age 83.7 years		T1 Discharge					
	56.6% female, AGU, UH							
	**Control** (n=202)	Conventional treatment: medical treatment, nursing care, physical therapy, and social assistance according with the usual practice of the unit.		36.7% improved ≥ 10 BI points		T1 11.9	8.7	
	Mean age 83.3 years							
	56.9% female, AGU, UH							
Blanc-Bisson 2008 [18]	**Intervention** (n=38)	Usual care plus early intensive physical therapy program: start day 1-2, strength training twice a day half an hour, 5 days a week until T1	T0 Admission	Mean Katz index	NA	T1 5.3	T1 12.6	NA
	Mean age 85.5 years		T1 Clinical stability	T0 6.7		T2 7.9		
	66% female, AGU, UH		T2 1 month after clinical stability	T1 5.3				
				T2 4.5				
	**Control** (n=38)	Usual care: transferred to arm-chair asap. Start day 3-6 walking 3 times a week with human help or without assistance. Physical therapy at home for 1 month		Mean Katz index		T1 5.3	T1 12.6	
	Mean age 85.4 years			T0 6.0		T1 5.3		
	79% female, AGU, UH			T1 4.7				
				T2 3.0				
Courtney 2009 [20,21]	**Intervention** (n=64) †	Individual exercise program and nursing visits, performed daily or several times a week. The intervention continued at home with home visits and regular telephone follow-up by a nurse.	T0 Admission	ADL: Mean score	WIQ distance^*^	T1 1.6	4.6	NA
	Mean age 78.1 years		T1 4 weeks post-discharge	index of ADL^*^	T0 23.54	T2 3.1		
	62% female, MU, CH		T2 12 weeks post-discharge	T0 0.36	T1 53.62	T3 3.1		
			T3 24 weeks post-discharge	T1 0.07	T2 54.83			
				T2 0.18	T3 62.89			
				T3 0.16	WIQ speed^*^			
				IADL: Mean IADL scale^*^	T0 16.21			
				T0 2.16	T1 41.30			
				T1 1.47	T2 44.62			
				T2 1.27	T3 48.56			
				T3 1.13	WIQ stairs^*^			
					T0 27.70			
					T1 46.73			
					T2 51.23			
					T3 57.20			
	**Control** (n=64) †	Routine care, discharge planning and rehabilitation advice. If necessary, in-home follow-up.		ADL: Mean score index of ADL^*^	WIQ distance^*^	T1 4.7	4.7	
	Mean age 79.4 years			T0 0.35	T0 20.22	T2 4.7		
	63% female, MU, CH			T1 0.69	T1 28.90	T3 4.7		
				T2 0.75	T2 21.59			
				T3 1.27	T3 19.93			
				IADL: Mean IADL scale^*^	WIQ speed^*^			
				T0 2.62	T0 14.43			
				T1 3.29	T1 22.09			
				T2 3.56	T2 17.89			
				T3 4.33	T3 16.58			
					WIQ stairs^*^			
					T0 24.12			
					T1 26.06			
					T2 24.40			
					T3 22.18			
De Morton 2007 [22]	**Intervention** (n=110) †	Usual care plus an individual exercise program. Twice daily, 5 days a week, for 20-30 minutes.	T0 Admission	Mean BI:	Mean TUG (s):	T1 1.8	5.0	T1 18.2
	Mean age 78 years		T1 Discharge	T0 66	T0 35			
	54% female, MU, ACH			T1 79	T1 36			
					Mean FAC:			
					T0 4.0			
					T1 4.8			
	**Control** (n=126) †	Usual care: daily medical assessment, 24 hour nursing assistance, and allied health service on referral from medical, nursing or other allied health staff.		Mean BI:	Mean TUG (s):	T1 1.6	6.0	T1 20.6
	Mean age 80 years			T0 68	T0 30			
	56% female, MU, ACH			T1 75	T1 26			
					Mean FAC:			
					T0 3.9			
					T1 4.7			
Jones 2006 [23]	**Intervention** (n=80) †	Usual care plus an individual exercise program Twice daily for approximately 30 minutes.	T0 Admission T1 Discharge	Mean change mBI: 11 points	Mean change TUG: 5.4 sec^*^	T1 5.0	9	T1 32.5
	Mean age 81.9 years							
	54% female, MU, ACH							
	**Control** (n=80) †	Usual care: medical, nursing and allied health intervention and discharge planning consistent with the patient’s diagnosis and resources available on the acute general medical wards.		Mean change mBI: 9 points	Mean change TUG: 1.2 sec^*^	T1 2.5	11	T1 51.3
	Mean age 82.9 years							
	61% female, MU, ACH							
Laver 2012 [14]	**Intervention** (n=22)	Individual interactive video game program (Wii Fit) 25 min/day, 5 days/week supervised by a physiotherapist	T0 Admission	IADL	TUG	T1 0	12.3	NA
	Mean age 85.2 years		T1 Discharge	T0 181	T0 38			
	86% female GU, ACH			T1 205	T1 28			
	**Control** (n=22)	Conventional physiotherapy, matching the patients abilities and treatment needs 25 min/day, 5 days/week		IADL	TUG	T1 0	14.95	NA
	Mean age 84.6 years			T0 141	T0 35			
	73% female GU, ACH			T1 190	T1 29			
Nikolaus 1999 [25]	**Intervention with follow-up** (n=181) †	In-hospital and post-discharge follow-up treatment by an interdisciplinary team. Physical or occupational therapy twice a week up to twice a day for 30 min	T0 Admission	Mean BI score:	NA	T2 18.2	33.5^*^	T1 4.4^*^
	Mean age 81.4 years		T1 Discharge	T0 71.0				T2 16.6
	Female 73.4%, GU, UH		T2 1 year post-discharge	T1 91.8				
				T2 81.2				
				Mean LB score:				
				T1 5.7				
				T2 5.6^*^				
	**Intervention without follow-up** (n=179) †	In-hospital treatment by an interdisciplinary team, followed by usual care at home		Mean score BI:		T2 16.8	40.7^*^	T1 7.3^*^
	Mean age 81.4 years			T0 71.0				T2 18.4
	Female 73.4%, GU, UH			T1 92.6				
				T2 82.3				
				Mean LB score:				
				T1 5.5				
				T2 4.1^*^				
	**Control** (n=185) †	Usual care in hospital		Mean score BI:		T2 17.3	42.7^*^	T1 8.1^*^
	Mean age 81.4 years			T0 71.0				T2 22.7
	Female 73.4%, GU, UH			T1 91.1				
				T2 80.9				
				Mean LB score:				
				T1 5.5				
				T2 4.3				
Siebens 2000 [28]	**Intervention** (n=149) †	Hospital-based exercise program twice a day. Encouragement to continue the program at home	T0 2 weeks pre-admission	Mean number of independent IADL	Independent walking	T1 6.7	12.0	NA
	Mean age 78.2 years		T1 1 month post-discharge	T0 5.3	T0 59.7%			
	62% female, ACH			T1 5.1^*^	T1 64.2%			
	**Control** (n=151) †	Usual care		Mean number of IADL independence	Independent walking	T1 6.6	10.5	
	Mean age 78.5 years			T0 5.3	T0 50.3%			
	59% female, ACH			T1 4.6^*^	T1 65.5%			

† = included only community dwelling old adults; ADL= Activities of Daily Living; IADL= Instrumental Activities of Daily Living; ICF=Intramural Care Facility; AGU=Acute Geriatric Unit; GU=Geriatric Unit; MU=Medical Unit; UH=University Hospital; CH=City Hospital; ACH=Acute Care Hospital; * = significant (p > 0.05); PPME=Physical Performance and Mobility Examination; WIQ=Walking Impairment Questionnaire; TUG=Timed Up and Go; FAC=Functional ambulation classification; NA=not available.

### Setting and intervention

Table 2 gives an overview of the settings and interventions of each included randomized controlled trial. The studies were performed on acute geriatric units, geriatric units and medical units of university hospitals, city hospitals and acute care hospitals. Early physical rehabilitation programs could be divided into two categories, (1) multidisciplinary programs with an exercise component and (2) usual care with an additional exercise program. In the review we refer to these categories as multidisciplinary programs and exercise programs, respectively. The main aim of the multidisciplinary programs was to maintain or obtain independent ADL and encourage returning home. Multidisciplinary intervention teams usually consisted of a geriatrician, (geriatric) nurses, social workers, physical therapists and occupational therapists [17,19,24,26,29]. The specific exercise component of the multidisciplinary intervention studies is hardly described, and information on intensity, duration, and frequency of exercises is often lacking. Usual care with an additional exercise program was provided in eight studies [14,16,18,20,22,23,25,28]. The patients in the exercise programs were supervised by allied health assistants, a physiotherapist or an occupational therapist. Patients performed exercises five times a week up to twice daily. The aim of the exercise programs was predominantly to improving functional outcomes by training strength, mobility, and balance. Strength exercises were progressed by increasing the number of sets and repetitions and walking exercises were progressed in intensity (from slow to moderate pace) or duration (from 5 to 30 minutes). In one study an interactive video gaming program was used to exercise. A Nintendo Wii fit group trained 25 min/day, 5 days/week under supervision of a physical therapist [14]. Some exercise programs supported participants to continue exercise after discharge [20,25,28]. This was achieved by educational materials, by (two or more regular) encouraging phone calls and home visits [20,28], or by a follow-up treatment, twice a week up to twice a day, including physical and occupational therapy [25]. The control groups of the studies generally received usual care according to the general routines of the hospital they were admitted to.

### Physical functioning

All included studies provided at least one outcome measure related to physical functioning. Most studies used measures of activities of daily living (ADL) and physical performance to describe physical functioning. Additional information about length of stay and discharge destination were also documented.

#### *Activities of daily living*

The included studies gained information on patient’s ADL by conducting interviews and (self-administered) questionnaires. Table 3 gives on overview of the used instruments to measure (I)ADL. Eleven of the thirteen studies reported the effects of their intervention on (I)ADL at time of hospital discharge [14,16-19,21-25,29]. Two multidisciplinary programs found that patients in exercise group compared to the patients in usual care group significantly improved more and worsened less in the number of basic ADL activities they were able to perform [24,29]. The other studies did not find a significant group difference in ADL at time of discharge. However, one study with an exercise intervention found a significant interaction effect between group and admission scores on the modified Barthel index meaning that patients with a low admission score who received an exercise intervention showed greater improvement in ADL than those patients who received only usual care [23]. Two studies reported a ceiling effect for the Barthel index and a floor effect for the Katz index [22,23]. Remaining studies did not found a significant difference on ADL between early physical rehabilitation programs and usual care (see Table 2).

**Table 3:** Overview of the instruments used to measure (I)ADL and physical performance

**Instrument**	**Study**	**Assessment**	**Score**		
			**Min**	**Max**	**Interpretation**
Katz index/ADL index	Courtney [20]	Ability to perform: Bathing, eating, dressing, continence, transfer to toilets and locomotion	0	6	0 independent - 6 dependent
	Bizon-Blanc [18]		0	12	0 independent - 12 dependent
	Counsell and Landefeld [19,24]	Ability to perform: Bathing, dressing, using the toilet, moving from a bed to a chair, and eating	0	5	0 independent – 5 dependent
(modified) Barthel index	Asplund and Salvedt [17,26]	Feeding, urinary and faecal continence, personal toilet, dressing, toilet use, transferring, walking outdoors, climbing stairs and bathing	0	20	1 dependent – 20 independent
	Abizanda, Jones, De Morton, Nicolaus [16,22,23,25]		0	100	0 dependent - 100 independent
Lawton index/IADL index	Nikolaus and Salvedt [25,26]	Ability to use telephone, shopping, food preparation, housekeeping, laundry, mode of transportation, responsibility for medication and ability to handle finances	0	8	0 dependent - 8 independent
	Counsell, Courtney, Landefeld and Siebens [19,20,24,28]	Ability to use telephone, shopping, food preparation, housekeeping, mode of transportation, responsibility for medication and ability to handle finances	0	7	0 dependent - 7 independent
Timed IADL	Laver [14]	The time needed to complete tasks addressing five IADL domains:			Lower scores indicates greater ability
		(1) communication, (2) finance, (3) cooking, (4) shopping, and (5) medicine.			
SIVIS dependency scales	Sleats [29]	SIVIS independency scale: 20 questions relating to orientation, communication, mobility, transfers, ADL, continence, catheter use, and decubitus			NA
Functional Independence Measure (FIM)	Laver and Siebens [14,28]	Measures the level of a patient’s disability and indicates how much assistance is required for the individual to carry out activities of daily living: Eating, Grooming, Bathing, Upper and lower body dressing, Toileting, Bladder and bowel management, Bed to chair transfer, Toilet and shower transfer, Locomotion, Stairs, Cognitive comprehension, Expression, Social interaction, Problem solving, Memory	18	126	18 dependent – 126 independent
Walking Impairment Questionnaire (WIQ)	Courtney [20]	Walking distance, walking speed and climbing stairs	0	100	Higher scores indicates greater ability
Timed Up and Go (TUG)	Jones, Laver, De Morton [14,22,23]	Time taken for the patient to rise from a chair, walk 3 m, turn and walk back to the chair			Lower scores indicates greater ability
Functional Ambulation Classification	De Morton [22]	Ability to ambulate over a 10 foot distance and 4 m length of foam	0	6	0 dependent - 6 independent
Physical activity scale	Siebens [28]	Questionnaire about walking ¼ mile, walking up 10 steps, crouching/kneeling, lifting/carrying 10 lbs			NA
Mobility	Counsell [19]	Walking to a table, walking inside the house, walking a block, walking uphill or upstairs, and running a short distance			NA
Physical Performance and Mobility Examination	Counsell [19]	Bed mobility, transfer skills, multiple stands, standing balance, step up one step and timed 6 m walk	0	6	0 dependent – 6 independent
Short Physical Performance Battery	Laver [14]	Three standing balance measures (tandem, semi-tandem, and side-by-side stands), five continuous chair stands, and a 2.44-meter walk.	0	12	0 dependent – 12 independent
Modified Berg Balance Scale	Laver [14]				NA

NA=Not available.

Of the eight studies that reported follow-up data on (I)ADL [17-20,24-26,28] only the three studies with an exercise intervention that provided patients with follow-up treatment after hospital discharge reported larger improvements in the exercise group than in the usual care group after 1, 6 and 12 months [20,25,28]. One study that did not provide patients with follow-up treatment also found a positive effect of the intervention on ADL at 3 months post-discharge. However, this study included patients who died by assigning them a score of zero, whereas the mortality rate was higher in the usual care group than the multidisciplinary group [26].

#### Physical performance

Seven studies reported measures of physical performance; Table 3 gives an overview of the used measurement instruments [14,19,20,22,23,28,29]. Three (2 multidisciplinary programs and 1 exercise programs) of the five studies describing physical performance at time of admission and discharge found that the intervention groups improved more or declined less than the usual care groups [19,23,29]. However, for one study the difference in the Timed Up and Go (TUG) was not significant after adjusting for confounders such as patient characteristics, admission modified Barthel Index, comorbidity, and mental scores [23].

For the TUG a floor effect was found. In one study, almost 40% of the patients were physically unable to perform the TUG at both admission and discharge [23], whereas in another study 23% of all patients were unable to perform the TUG at admission to the hospital [22].

Two studies reported follow-up results on physical performance, but the results are ambiguous [20,28]. One study [28] reported no significant difference between the patients in the exercise group and in the usual care group in the change in physical performance scores obtained 2 weeks before admission and one month after discharge. Conversely, the other study [20] reports that the exercise group had greater improvement over time (up to 24 weeks after discharge) in walking distance, walking speed and stair climbing. Similar contradicting results were found on more general outcome measures of physical functioning, provided by a health questionnaire that contained physical well-being and physical activity. No group differences were found for the National Health Survey Physical Activity Scale at one month follow-up [28], while the other study found that the exercise group scored higher than the usual care group on physical health related quality of life at 4, 12, and 24 weeks after discharge [21]. In short, follow-up results on physical performance are contradictory. Nevertheless, at time of discharge most studies show a greater improvement for patients in the multidisciplinary and exercise group than for the patients in the usual care group.

#### Length of stay and discharge destination

All included randomized controlled trials reported about the length of stay, which varied between the 4.7 days and 42.7 days. Four studies (three multidisciplinary programs) reported a significant difference shorter length of stay of the exercise group than for the usual care group [17,25,27,29].

From the seven studies [17,19,22-25,29] that reported on discharge destination, five studies found that a higher proportion of patients in the early physical rehabilitation group than in the usual care group were discharged home, instead of being transferred to additional (sub-acute) hospital treatment or to institutionalized care, however, results were significant in three studies (Table 2). Two multidisciplinary programs found that respectively 14% and 18% of the patients in the multidisciplinary group were discharged to a long-term care institution opposed to 22% and 27% of usual care patients [24,29]. The third study, an exercise intervention, included only patients who had lived at home before admission and found that 4% of the patients in the exercise group and 8% of the patients in the usual care group were discharged to long-term care institutions [25].

### Feasibility

Feasibility of early physical rehabilitation programs, was explicitly assessed in four studies (Table 4) comparing usual care with an exercise intervention program [14,30-32]. In addition included randomized controlled trails with an exercise program reported on enrolment (n=3), adherence rate (n=2) and adverse events (n=5), while multidisciplinary studies reported on enrolment (n=3) only.

**Table 4:** Setting and study characteristics feasibility

**Study**	**Population & setting**	**Intervention**	**Feasibility**
Brown 2006 [30]	**Intervention** (n=7)	Exercise sessions twice a day, 7 days a week. After discharge 20-30 min walk each day and resistance exercise every other day	n=605 admitted, n=76 included
	Mean age 70.2 years, 0% female		n=66 declined participation
	**Control** (n=2)	Usual care which included physical therapy if a consult was initiated by the physician	
	Mean age 70.2 years, 0% female		
Mallery 2003 [31]	**Intervention** (n=19)	Usual care plus resistance exercise 3 times per week, 30-40 min, assisted by a physiotherapist	n=395 admitted, n=39 included
	Mean age 82.7 years		Participation 71%,
	74% female, GU, UH		Adherence 63%
			No adverse events
	**Control** (n=20)	Usual care plus passive range of motion training 3 times per week, 30-40 min, assisted by a physiotherapist	Participation 96%,
	Mean age 81.4 years		Adherence 95%
	45% female, GU, UH		No adverse events
Nolan 2008 [32]	**Intervention** (n=196)	Participated in the Functional Maintenance Exercise Program, 6 times per week, 30 min	n=1021 admitted, n= 220 included
	Mean age 83.6 years,		33 withdrawn
	68% female, GU, UH		
	**Control** (n=24)	Usual care with usual physiotherapy	
	Mean age 85.4 years		
	67% female, GU, UH		
Laver 2012 [14]	**Intervention** (n=22)	Individual interactive video game program (Wii Fit) 25 min/day, 5 days/week supervised by a physiotherapist	n=235 admitted, n=44 included
	Mean age 85.2 years		90% adherence rate
	86% female GU, ACH		No adverse events
	**Control** (n=22)	Conventional physiotherapy, matching the patients abilities and treatment needs 25 min/day, 5 days/week	91% adherence rate
	Mean age 84.6 years		1 adverse event, conscious collapse
	73% female GU, ACH		

GU=Geriatric Unit; UH=University Hospital.

Subjects enrolled in the studies explicitly assessing feasibility were respectively above the age of 60 or 70. The most common exclusion criteria were severe impairments in physical performance and cognition, requiring palliative care, expected short length of stay, and medical instability. One study specifically targeted frail patients that were at risk of functional decline [32].

Feasibility was measured quantifying patients enrolment, patient adherence to the program and at patient’s safety in the context of the exercise program. Nine of the 11 studies found that between the 14% and 48% of the admitted patients met the inclusion to be enrolled in the programs, and between 3 and 19% of the patients were not willing to participate [14,17,19,22-24,28,31,32]. In general patients not willing to participate stated that they did not feel like exercising or that they did not believe they could exercise. They felt unwell and/or were scared that exercising would make them feel worse.

In one study [30] only 2% of all admitted patients were included. Of the 76 patients that met the inclusion criteria only 10 consented to participating while 87% of the eligible patients refused to take part in the program. In fact none of the included patients were able to start with the exercise program. Reasons for not participating in the exercise program were not of a physical nature. Instead, patients were discharged before therapy could begin or were unavailable due to diagnostic tests or appointments with healthcare professionals.

Adherence rates were fairly high between the 60% and 90% [14,20,31,32]. The most common reasons for dropping out of the intervention programs were early discharge, being transferred to intensive or palliative care, being medically unstable, and death [17-19,22,23,28,32].

A final measure of feasibility is patient safety during the exercise program. One feasibility study and six RCT studies included in this review reported on potential side effects such as injuries, accidents, and more specifically, fall incidents, related to participating in the early physical rehabilitation programs [14,16,18,20,23,28,31]. None of the studies found any differences in the number of incidents between the exercise groups and usual care groups.

## Discussion

The first aim of this review was to evaluate the effect of early physical rehabilitation programs for geriatric hospitalized patients on physical functioning. A total of 15 articles, reporting on 13 studies, were included that provided early physical rehabilitation programs in a hospital setting. The present review shows that early physical rehabilitation programs might be beneficial to prevent rapid decline in physical functioning.

A classification could be made between multidisciplinary programs with an exercise component and usual care with an exercise component. Multidisciplinary programs reduced the length of stay for patients in the exercise groups compared to the patients receiving usual care. Additionally, the patients in the multidisciplinary programs were less likely to be discharged to a nursing home or other forms of institutionalized care than patients in the usual care group. The usual care programs with an exercise intervention had the main aim to improve functional outcomes, some of the studies showed indeed an improvement on ADL and physical performance.

The two types of exercise interventions, e.g. multidisciplinary programs with an exercise component and usual care with an exercise component, did not find different results in physical functioning at time of discharge. At time of discharge, results on physical performance were to some extent contradicting, but the majority of the included studies showed that patients in the exercise groups had better ADL and physical performance than patients in the usual care groups, although those results were not always significant. Follow-up results on ADL and physical performance showed that persistent positive effects were mainly found in studies that provided patients with continuous interventions after hospital discharge. The studies that provided patients with only in-hospital exercise interventions found little or no effect on ADL and physical performance at follow-up examinations. These results are in line with other studies that investigate the effects of care in geriatric units and of inpatient rehabilitation for geriatric patients on functional parameters [10,33]. The effects of only in-hospital interventions were clearly positive at time of discharge but were greatly reduced during follow-up. These results suggest that the recovery of patients could further benefit from a community based or in-home intervention programs which build on in-hospital programs. Such programs could consist of physical or occupational therapy. In addition, the present review shows in home visits and follow-up telephone calls might be effective for adherence for home-based exercise [20]. Further research is needed to clarify the effects and feasibility of community based and in-home intervention programs in old adults after hospital discharge.

The studies presented in this review included very heterogeneous groups. Half of all studies excluded nursing home patients. Probably, due to the aim of those studies to facilitate discharge home. The studies in this review that targeted frail older patients, patients with increased risk for readmission or patients with a high risk for nursing home admission found positive effects of an exercise program on ADL, length of stay and discharge destination [20,25,26]. This result indicates that frail old adults and nursing home patients may benefit from early rehabilitation. Targeting the right population for early physical rehabilitation may be seen as crucial.

The outcomes with regard to physical functioning were measured by a variety of instruments and at different points in time, some of these instruments demonstrated floor and ceiling effects. Floor and ceiling effects could influence the outcomes and distort the results. There were also cases in which information could only be collected with the help of close relatives and caregivers, because relevant information could not be obtained from the patients themselves. The limitations of the current used measurement instruments implies that there might be a need for more sensitive instruments that measure aspects of physical functioning in hospitalized old adults.

Finally, the second aim of this review was to investigate the feasibility of early physical rehabilitation programs for acute ill older patients. The early physical rehabilitation programs must be safe to perform and may not cause high numbers of drop outs. There were four studies identified reporting on the feasibility of in-hospital exercise programs [14,30-32]. Additionally, a part of the randomized controlled trials, used to determine the effect on physical functioning, reported also on some feasibility points. Several studies included in this review affirmed the safety of early rehabilitation programs. Patients in the early physical rehabilitation groups were not more often injured, nor did they experience more adverse events or falls than usual care patients. Results on patient recruitment were contradictory and should be interpreted with care due to the limited number of available studies. Adherence rates were high for most studies. Patient as well as the intervention provider’s satisfaction was higher when patients were treated with a early physical rehabilitation program instead of usual care [19]. There was one study that encountered difficulties in recruiting patients and was unable to have any patients participate in the exercise program at all [30]. Patients refused to participate or were unavailable at scheduled time of therapy. One of the greatest barriers in the implementation of intervention research, according to the opinion of nurses, was a lack of awareness and knowledge [34]. Equally important in this regard were difficulties in the cooperation of interdisciplinary team members [34]. Education of staff and patients about the safety and the benefits of early physical rehabilitation as well as regular team conferences that improve coordination might help to increase the success rate of intervention programs with regard to participate.

## Limitations

The total number of included studies in this review is small and the methodological quality for most studies was moderate. Most articles scored particularly low on blinding the assessor, the care provider, and the participant. However, it may be regarded as almost impossible to assure blinding in the context of early physical rehabilitative interventions. One of the major limitations of the included studies lies with the poor descriptions of exercise in early physical rehabilitation programs. Since there is often no clear description on type, duration, frequency, and intensity of exercises that patients receive. Furthermore, because many multidisciplinary programs are focused on outcomes beyond functionality, e.g. early discharge planning, it is difficult to determine what the effect of the exercise component on physical functioning is in this setting. Future research should provide quantification of exercises that older patients perform during their hospital stay, so that there would be a clearer view on the dose-response relationship of physical activity and functional outcomes.

## Conclusion

Early physical rehabilitation programs for acutely hospitalized old adults have the potential to improve physical functioning, and also to prevent patients from being discharged to nursing homes or other forms of institutionalized care. Interventions including a follow-up program after hospital discharge increase the chance of maintaining positive effects on functionality for longer periods of time. Early physical rehabilitation for acutely hospitalized older adults seem to be safe to execute in terms of adverse events such as falls or other injuries, but recruiting the most suitable patients and getting them to participate regularly in the program can be a challenge. Therefore, the commitment and collaboration of staff is of great importance. Further research is needed to quantify the physical activity of patients in early physical rehabilitation programs and to determine the effects and feasibility of community-based and in-home exercise programs.

## Competing interests

The authors declare that they have no proprietary, financial, professional, or other personal competing interests of any nature or kind.

## Authors’ contribution

Nienke Kosse and Alisa Dutmer managed the review process and wrote the draft of the full manuscript together with Claudine Lamoth. All authors critically revised the manuscript and approved the final version.

## Pre-publication history

The pre-publication history for this paper can be accessed here:

http://www.biomedcentral.com/1471-2318/13/107/prepub
